# First steps towards sustainability? University freshmen perceptions on nature versus environment

**DOI:** 10.1371/journal.pone.0234560

**Published:** 2020-06-15

**Authors:** Michaela Maurer, Franz Xaver Bogner

**Affiliations:** Department of Biology Education, University of Bayreuth, Z-MNU (Centre of Math & Science Education), Bayreuth, Germany; University of Westminster, UNITED KINGDOM

## Abstract

The Global Earth Overshoot Day, the date when all annually available natural resources are consumed, is set for July this year. For densely populated European countries like Germany or Switzerland, that specific day is due even earlier (May). To overcome such an unsustainable lifestyle, immediate actions are required, which includes substantial educational efforts. As the model of "Sustainable Development" is complex, appropriate pedagogical actions need to support cognitive learning, critical thinking and behavioural actions. Knowledge about individual conceptions in relation to the *Environment*, *Nature* and *Ecological Footprints* contributes to pre-conditions to succeed. To what extent present teaching methods influenced individual conceptions during the first UN-decade regarding those terms is illustrated by 464 Swiss-German university freshmen who participated in our paper-pencil test, which is based on four open questions. The term of *Environment* was perceived as the sum of biocentric, ecocentric and anthropocentric views. The participants often equated the term to *Nature* and associated it with positive feelings or emotions. Therefore, calm, joy and aesthetic appreciation were predominantly named. Regardless of the concept, humans were perceived as the *Greatest Environmental Threat*. In contrast, recommendations to reduce *Environmental Footprints* regarding mobility & transport, waste avoidance and consumption differ. Following a binary logistic regression analysis, the involvement of the Inclusion of Self Scale (INS) was used as an explanatory variable to detect patterns of those conceptions. Relating sustainable concepts, natural resources were frequently named exceeding saving water and energy or other association dealt with second-hand issues or regional/ seasonal usages. Such ideas are shaped by experiences and scientific expertise.

## Introduction

### Transformation into a sustainable future

First environmental movements date back to the early 1970s, when pesticides were spreading uncontrollably around the globe. Habitats far away from any settlement, for instance, those of penguins and seals, were found contaminated although no one would have suspected it. In consequence, Carson’s *Silent Spring* became one of the first publications to raise awareness of environmental problems within the general public [1]. Fifty years later, young people still have to demonstrate for saving the planet. Monitoring anthropocentric influences and overusing natural resources is not unknown. The famous book *Club of Rome–The Limits to Growth* [2] has already identified resources such as soil, air, water and genetic diversity as most vulnerable [3]. The *Brundtland-report* [4] in the 1980s was an initial step to roadmap sustainability, urging “to keep options open for future generations, the present generation must begin now, and begin together, nationally and internationally”. All initiatives stressed the need for education beyond cognitive levels, affecting attitude and behaviour levels which may lead to appropriate action [5–9]. The Rio conference formulated Education for Sustainable Developments (ESD) requesting re-orientation first within Agenda 21 [10] and second with Agenda 2030 [11]. The three pillars: ecology, economy and social aspects are considered to impact individual awareness to tackle the environmental crisis. Observing young people’s perceptions of environment, nature, concern and willingness to act could help to understand our current development. Education for Sustainable Development (ESD) requires general education, innovative focus of learning (assessment, anticipatory and networked thinking), subjective experience (experience of nature), understanding for coherences (economy, ecology and social aspect) and ethics (understanding of values) that fosters environmental awareness and impacts environmental behaviour [12,13].

### Conceptions

Educational efforts are supposed to support thinking experiences, as learning processes are characterised by personal experiences and scientific explanations [14]. According to Piaget, for instance, individuals adapt their sum of knowledge during life, influenced by participation in social activities [15]. Approaching (subjectification) develops individual perceptions whereas withdrawing (professional objectivation) creates realities and perceptions [16]. Constructivism is the theory of knowledge in which learners are identifying constructs from a subject [17]. Radical constructivism forms memories and imagination in minds. Imaginations are important to interpreting individual surroundings of representation into individual world conceptions [18]. Relating to environmental issues, emotions and feelings (e.g. fears, joy) are compulsory [19] e.g. for sensitive topics like climate change or biodiversity loss [20]. However, besides scientific conceptions, alternative conceptions coexist (*e*.*g*. [21]). Educational background in this study was the most important determinant for increasing scientific knowledge when 6^th^ graders, 10^th^ graders and freshmen were asked about the perception of tree assimilation and wood synthesis (*N* = 885). Misconceptions in environmental education cause problems [22], especially if teachers are inexperienced or follow on their misconceptions [23]. Bonnett & Elliot [24] already pointed to a close relationship between natural environments and human beings. Psychological factors may explain why some people are more motivated to protect the environment [25] than others (*e*.*g*. intrinsic motivation to reduce consumption). Overall, several studies about teachers’ [26] and students’ conceptions point to complex processes [27]. Studies about environment versus nature, emotional perceptions and sustainable self-perceptions about environmental threats may help to reduce the footprint by detecting respective interrelations [28].

### Environmental ethic

The term *environmental ethic*, dating back to the 1970s, addresses aspects of environmental crises (*e*.*g*. forest dieback, acid rain, air pollution). It is part of the ethic discipline of normative appropriate and morally responsible interaction [29] with the natural world [3]. Environmental perception constructs an individual image of the world based on individual imagination [30]. This concept, as defined by Uexkúll [31], represents the exploration of living organisms of the outer world (called physiological environment). Thus, bacteria, fungi and other living organisms were neglected. Today, however, the term includes all living organisms that are part of the biospheric ecosystem [32,33]. Therefore, it includes all biotic and abiotic factors as well as the relationships between organisms. The fundamental question is, thereby, whether nature’s value is dependent on humans or has its in-made value [3,34] since nature is all that is not man-made. Two antagonistic views coexist:

- anthropocentric refers to human-beings utilising resources such as water, soil and air [35]. Within this context, protecting the environment depends on humans’ benefits (e.g. protecting honeybees for pollination and honey extraction), non-human organism or natural phenomena are of instrumental or aesthetic value [36],
- physiocentric is a generic term for a pathocentric, biocentric and ecocentric focus that humans have to respect. Pathocentric includes the ability to suffer (human and higher life forms) whereas biocentric represents nature and all living organisms with intrinsic values [37]. Two options are distinguished: egalitarian bio-centric ones, where all living organism have the same value or hierarchically modularised values, where all living organism have different values (scala naturae–from the bacterium to the human being). Ecocentric values including all elements of nature (biotic and abiotic) are equally represented *e*.*g*. animal and plant species, rivers or mountains, and even ecosystems according to Aldo Leopold and Arne Næss [38]. Efforts to protect eco-systems are subsumed under holism.

The value of biodiversity displays how closely ethics and the environment are interrelated [39]. Ecology simply provides the respective knowledge to understand the dynamics of biodiversity without necessarily including information about ethical values. In return, ethics is by far too vast a topic to explain this value without ecological knowledge. It is, however, indispensable to clarify responsibilities if the protection of pollinators is concerned. Observing different groups’ perceptions regarding the benefits and conservation of bees via semantic differentials, beekeepers’ displayed the highest interest, followed by university students and primary students [36]. Education is, thereby, the basis for attitudes/values and pro-environmental behaviour [40]. This has recently been demonstrated while assessing tenth graders (*N* = 275) regarding their perceptions of biodiversity [41]. Only one of three concepts was regularly identified (species diversity) whereas the others were only occasionally detected (genetic diversity and ecosystem diversity). A biodiversity module (Future Forest) obtained long-term knowledge gains by linking a citizen science project which aimed at engaging this cohort of students (*N* = 205) in biodiversity-related subjects [42].

However, it is known from literature that the connectedness of nature level operates positively with environmental behaviour and values [43,44]. Surprisingly, there is a lack of studies with young adults, which link concepts of the *Environment* or *Nature* linking to sustainable aspects. Leisure activities lead to the destruction of and alienation from nature [45]. It prompts lead open research questions regarding perceptions in comparison to the following.

### Research goals

Our main research goal was (i) to monitor freshmen´s conceptions about the environment; (ii) what kind of emotions/feeling they have towards nature; (iii) which notions of the most eminent environmental hazards exist and (iv) how freshmen present ideas to reduce their ecological footprint.

## Methods

### Ethics statement

According to the general ethical and scientific standards for research with humans, our paper-pencil test was in line with all required standards (HRA, Article 51, paragraph 2). Data like gender, age and study-status were recorded pseudo-anonymously.

### Sample

Our study included 464 Swiss German university freshmen from a wide range of study programs (*N* = 464, *M* = 21.3, *SD ±* 3.1, female = 66.5%). The Swiss population density is 216 people per square meter in 2018 [46]. As our paper-pencil test was used for another recent study [47], we compared both. We adapted the findings of the seven-point Likert scale (Inclusion of Nature in Self (INS)–Scale (”A = very low” to “G = very strong”) [48], with two overlapping circles labelled ‘self’ and ‘nature’ to show the relationship between the two of them.

### Categorisation

After extracting the main categories by applying the qualitative content analysis of Mayring [49], our study was based on four fields:

- perceptions about *environment*, where we used 14 categories inductively that we separated into three main categories: anthropocentric (“I live/surround me”, “anthropocentric influence”), biocentric (“animals”, “plants”, “organism”, “environmental protection”, “human”) and ecocentric (“abiotic”, “planet earth”, “ecosystem”, “habitat”, “interaction between organism”, “we live/surrounds us”, “nature”),
- *emotions and feelings* connected with *nature*, where we used 43 sub-categories inductively concerning to ten main categories (“admiration”, “anger”, “anxiety”, “aesthetic appreciation”, “calmness”, “disgust”, “fear”, “joy”, “sadness” and “shame”) (Table 1),
- *greatest environmental hazard*, where we allocated 19 sub-categories and
- *reducing the ecological footprint*, where we allocated 21 sub-categories for five identical main categories (“awareness”, “mobility & transport”, organism”, resource & consumption” and “waste”) (Table 1).

**Table 1:** Coding guidelines for the main categories of freshmen´s perception.

Categories of conceptions	Definition	Examples
Anthropocentrism (a)	Humans being in the centre of their perspective on nature	Pollutant uptake, the environment that surrounds me
Biocentrism (a)	All living things, including plants and animals	Human, animal plant, organism
Ecocentrism (a, c)	Nature being in the centre and mean views are solely needs	Ecosystem, river, environment that surround us
Admiration (b)	The feeling or description of admiring something	Fascination (*e*.*g*. nature), respect (*e*.*g*. natural forces)
Anger (b)	A strong feeling that makes you unpleasant because something unfair happens	frustration, brutality against nature
Anxiety (b)	An uncomfortable feeling of worry about something that is happening or might happen in the future	not take care of nature, dependence
Aesthetic Appreciation (b)	Include an aesthetic appreciation of the objects or powerfully description based on nature for instance (= aesthetic emotion meaning)	aesthetics, unspoiled landscape
Calmness (b)	A peaceful, quiet or relaxed state without hurried movement or noise	free, freedom, silence, relaxation
Disgust (b)	A strong feeling of disapproval and dislike against something, *e*.*g*. an organism	disgust for animals, birds
Fear (b)	An unpleasant emotion or thought that occurs when you are frightened or worried.	fear of the destruction, cryophobia, less food
Joy (b)	A memory or thing that causes happiness or connectedness to nature	hobby, time off, luck, satisfaction
Sadness (b)	A feeling of being sad or unhappy	the destruction caused by human activities
Shame (b)	An uncomfortable feeling of guilt	feelings of guilt, charm
Awareness (c)	Knowledge or perception of a situation or fact	human interference (environmental hazard) versus conscious behaviour (ecological footprint-reverse)
Resources & Consumption (c)	Consumption behaviour of non-renewable, or less often, renewable resources and consumption of goods	energy and water consumption, overproduction (environmental hazard) versus preferring regional and seasonal products (ecological footprint-reverse)
Mobility & Transport (c)	Mobile transportation, used for transporting people or goods on land, especially on roads	increasing mobility (environmental hazard) versus limiting mobility and using alternatives *e*.*g*. public transport (ecological footprint-reverse)
Waste (c)	End products, resulting from private households or industry	plastic, waste (environmental hazard) versus avoidance of disposable packaging (ecological footprint-reverse)

Freshmen perceptions based on open questions belonging to the categories for *environmental ethics* (a), *emotions and feelings* (b) and the *greatest environmental hazard* relating to their ecological footprint (c) (retrieved and adapted from the Cambridge dictionary).

To assess 2620 statements, 15% of all data were randomly selected after six months from the first author (inter-rater reliability) and a second nonpartisan person (intra-rater reliability) to test the quality (Table 2).

**Table 2:** Cohen’s kappa scores for inter- and intra-reliability.

	Cohens-Cappa	
Questions:	Interrater reliability	Intrarater reliability
(i) How do freshmen’s perceive their environment?	0.70	0,55
(ii) What kind of emotions/feelings do they connect with nature?	0.91	0.70
(iii) Which notions of the greatest environmental hazards do they have?	0.75	0.52
(iv) How can freshmen reduce their ecological footprint?	0.67	0.61

According to literature, Cohen’s kappa scored almost perfectly above 0.75 and substantial above 0.60. The values of zero, a randomly correlation is assumed [50]. The resulting Cohen’s kappa scores indicate an overall open questions a good level of agreement between the raters (Table 2).

### Data analyses

All statistical tests were analysed using R (The R Foundation for Statistical Computing for Windows Version 3.6.0; www.r-project.org). To explore the general concepts, we applied Ward.D2 hierarchical cluster analysis (package pvclust; for method, see [51]) based on multiscale bootstrap resampling. It provides p-values that in line with the data. Furthermore, we used binary logistic regression analysis to examine the effects between the main categories (observed = one, not observed = zero) following the categorical variables through the Inclusion of Nature in Self (INS)–Scale [48]. For the contingency analysis C_corr_, we set a limit of 0.2 and a significance level of α = 0.001.

## Results

We formed all categories inductively from open questions (definitions, see Table 1). Some examples are displayed in Table 3.

**Table 3:** Categorisation examples from freshmen perceptions of the *environment*.

		Main categories		
ID	Statements	Anthropho-centric^1^	Biocentric^2^	Ecocentric^3^
78	Everything that **surrounds me**^1^: **nature**^3^ as well as **animals**^2^ and **humans**^2^	1	1	1
80	Everything that **surrounds me**^1^ as a **human**^2^ outside of my personality. **friends**^3^, **family**^3^, **nature**^3^	1	1	1
88	**Abiotic**^3^ and **biotic**^2^ world, where **I live**^1^	1	1	1
253	**Nature**^3^, the **world**^3^ in which **we live**^3^	0	0	1

## Environment

**Fig 1:**
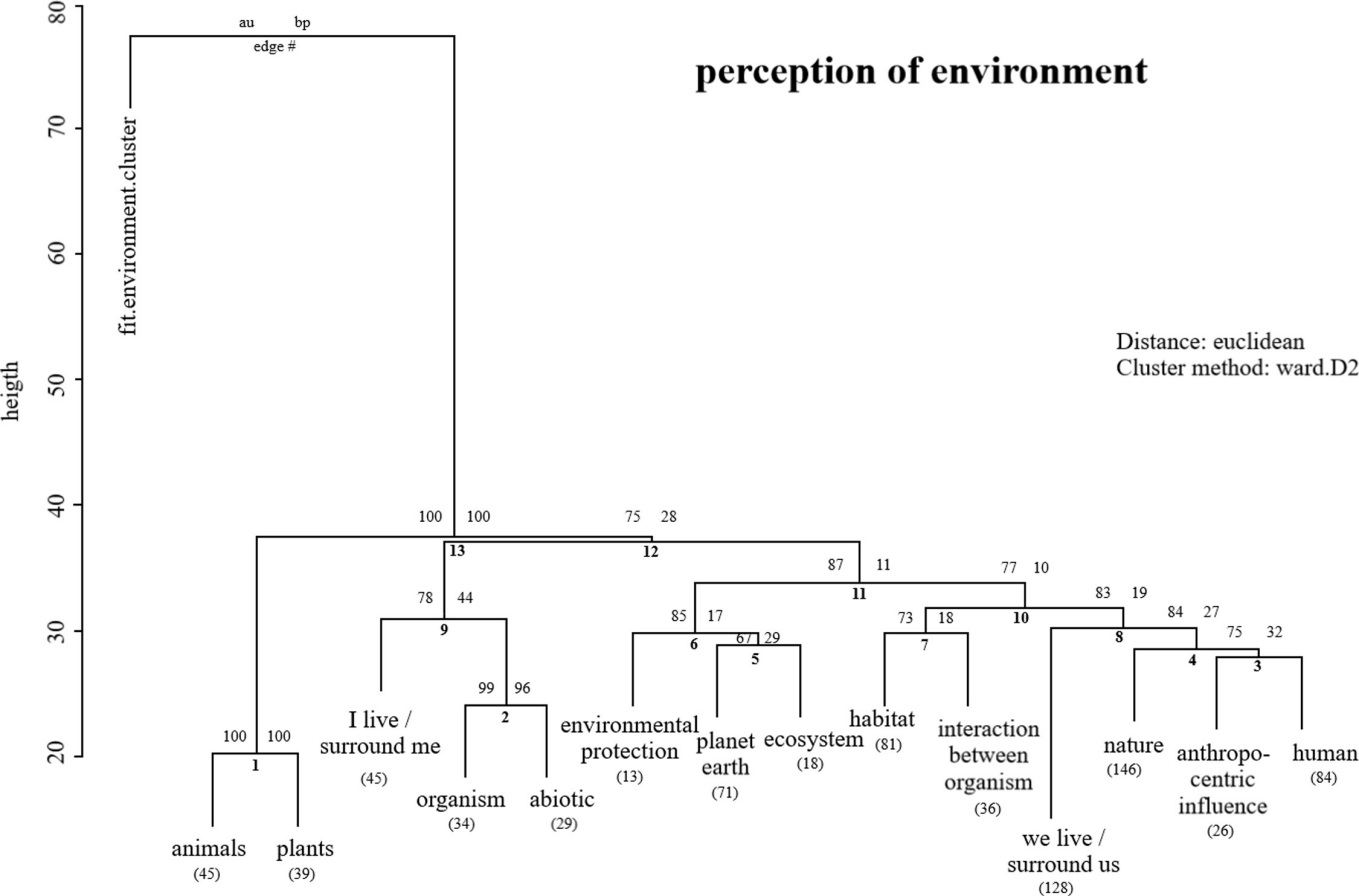
Cluster dendrogram with p-values based on the freshmen’s perception of the *environment*. Numbers above the branches on the right: standard bootstrap p-value and on the left illustrate approximately unbiased (AU) p-values (Clusters with AU > = 95% are indicated by the rectangles and are considered to be strongly supported our data). Numbers in brackets below the categories are the observation of all participants.

321 freshmen responded to the question of how they perceive the term *environment*: five of one participant yielded perceptions of either anthropocentric, biocentric or ecocentric (see methods). The conceptual patterns of 14 sub-categories do not follow a certain environmental ethical view (anthropocentrism, biocentrism and ecocentrism, see method) as one branch [51] (Fig 1). Students see themselves rather as a part of the environment (*N* = 128, sub-category “we live/surround us”) in the centre of the environment (*N* = 45, “I live/surround me). Some concepts were observed on one branch (*e*.*g*. animals and plants).

### Emotions and feelings to nature

**Fig 2:**
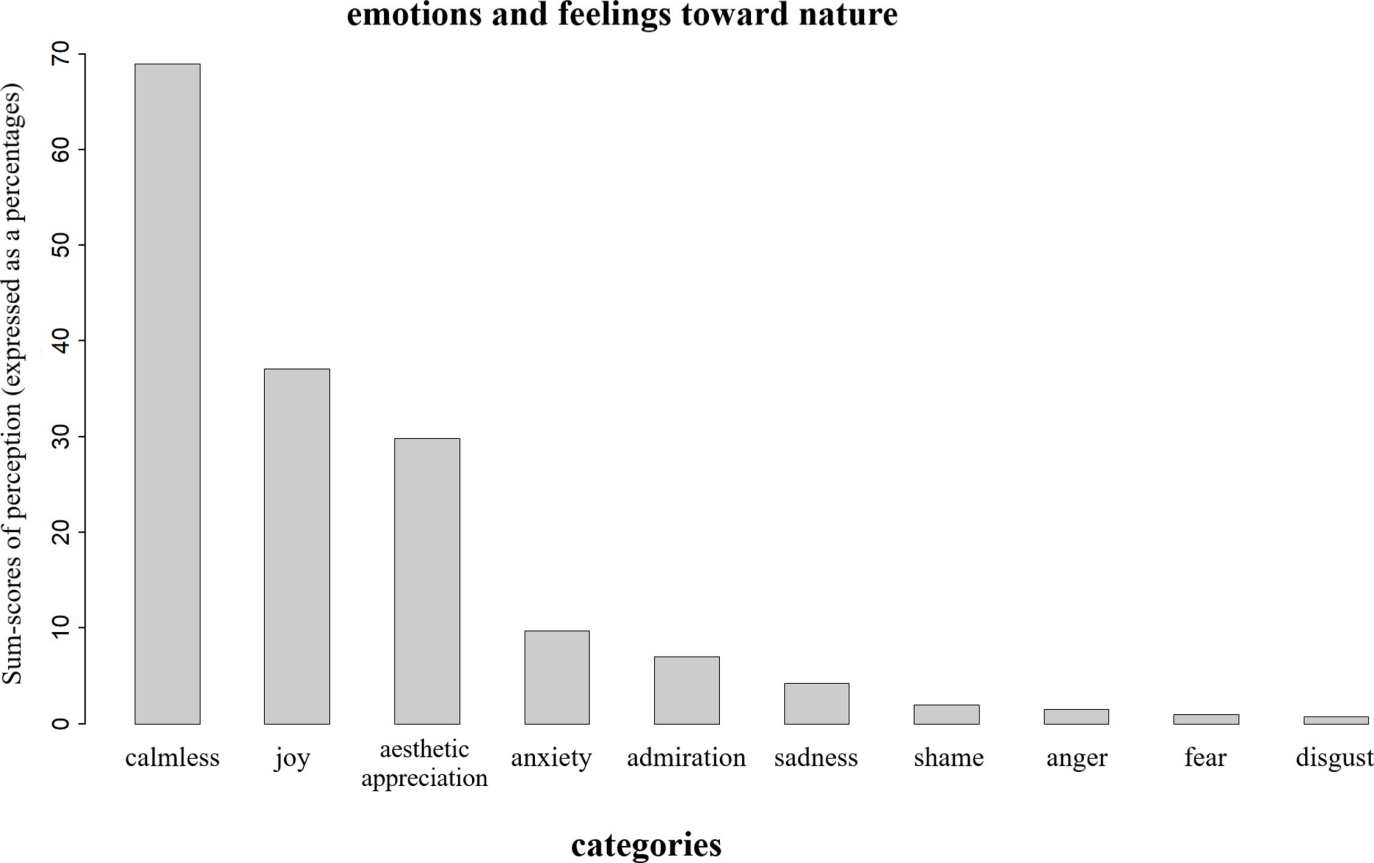
Reflection of freshmen (*N* = 402) perception following emotions and feelings.

Freshmen (*N* = 402) associate *emotions and feelings* with nature as a variety of different perceptions belonging to ten categories (Fig 2). Nature was mainly connected to positive feelings and emotions. The connectedness to nature as an explanatory variable explained no difference between the categories of the three main observations (*e*.*g*. joy: *𝛽*_*intercep*_ = -0.97, *SD* ± 0.39, *z-value* = -2. 52, *p* = 0.001, odds ratio = 0.93). Nature stands predominantly for human welfare like freedom, silence and private activities outside. The negative trend was less evident (*e*.*g*. sadness of the destruction of nature or disgust for particular species).

The relationship between nature and the self was analysed via the Inclusion of Nature in Self scale (INS) in an earlier study of the same participants (*M* = 3.954, *SD* ± 1.15) [47]. We used those results in the following as the independent variable and the binary category as depend variable. A binary logistic regression analysis [family = binomial ("logit")] delivered different outcomes between the categories regarding perceptions of the biggest environment hazard and reducing the ecological footprint (Table 4).

**Table 4:** Binary logistic regression of coefficients regarding emotions.

Category	𝛽_intercept_	SD	z-value	*Pr(>|z|)*	eβ
Admiration	1.30	±0.40	-3.31	>0.001	1.14
Joy	1.05	±0.37	-2.87	>0.001	1.09
Aesthetic appreciation	-0.97	±0.39	-2.52	0.01	0.99

eβ = Odds ratio

### Environmental hazard versus ecological footprint

**Fig 3:**
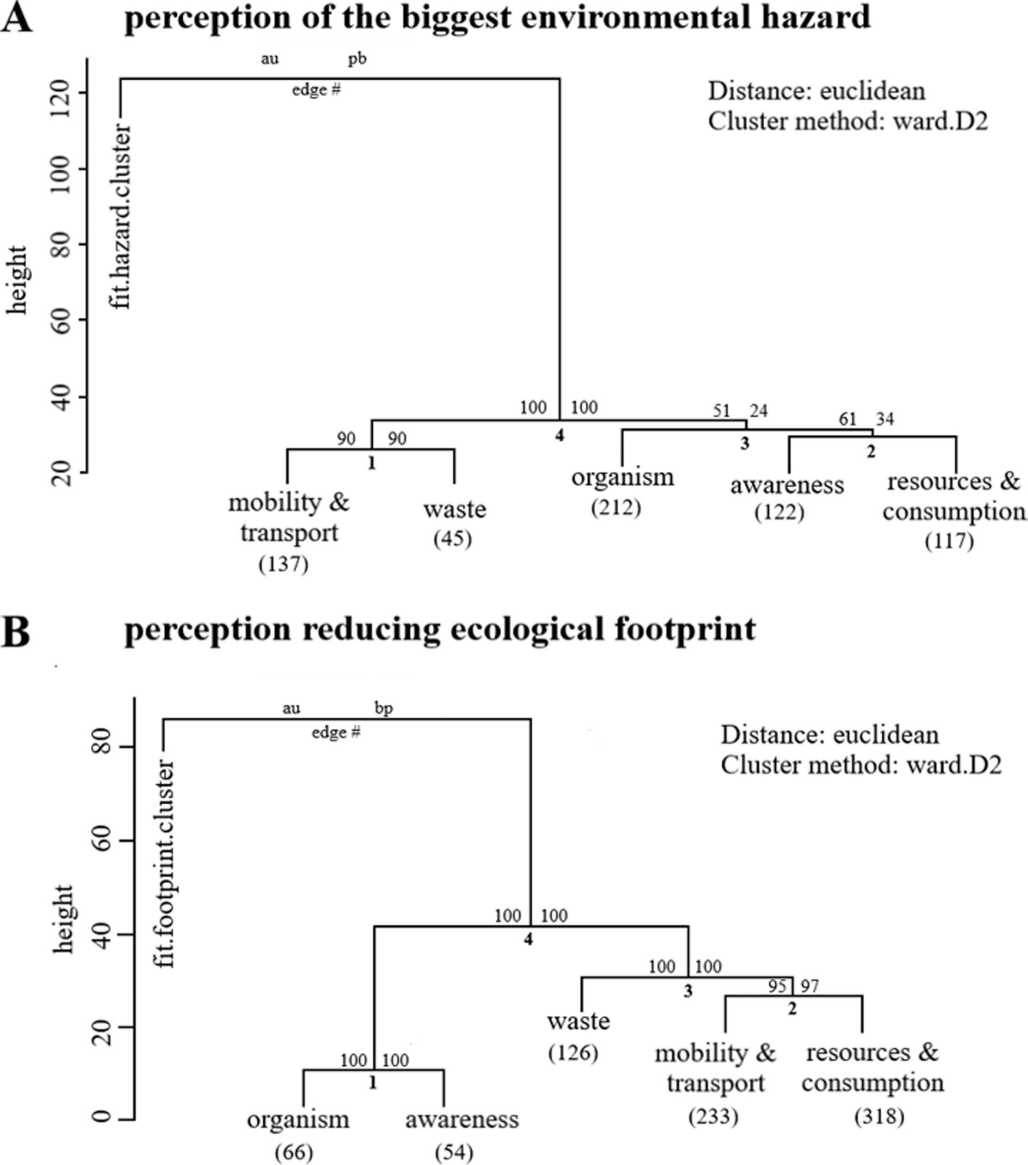
Cluster dendrogram with p-values based on the freshmen’s perception: (A) greatest environmental hazard (*N*_*participant’s*_ = 400) and (B) reducing ecological footprint (*N*_*participant’s*_ = 413). Numbers above the branches on the right: standard bootstrap p-value and on the left illustrate approximately unbiased (AU) p-values (Clusters with AU > = 95% are indicated by the rectangles and are considered to be strongly supported our data). Numbers in brackets below the categories are the observation of all participants.

A qualitative content analysis categorised the students’ ideas about the environmental hazard and reduction of their ecological footprint. We identified 1430 statements (*n*_*environmental hazard*_ = 633, *n*_*ecological footprint*_ = 797), which form five main categories (definitions, see Table 1). A contingency analysis showed a relationship of all categories between environmental hazards and reducing their ecological footprint (*C*_*corr*_ = 0.54, *n* = 1430, *p* < 0.001). Perceptions of reducing their ecological footprint are much higher of all categories in comparison to the perception of the greatest environmental hazard. A second analysis, a hierarchical cluster analysis, confirmed similarities that conceptions were not following the same clusters based on both questions (Fig 3).

A binary logistic regression analysis [family = binomial ("logit")] delivered different outcomes between the categories regarding perceptions of the biggest environment hazard and reducing ecological footprint (Table 5).

**Table 5:** Binary logistic regression of coefficients regarding environmental hazard^1^ and footprint^2^.

Category	𝛽_intercept_	SD	z-value	*Pr(>|z|)*	eβ (INS)
Awareness^1^	-1.34	±0.40	-3.41	>0.001	1.08
Awareness^2^	-1.72	±0.53	-3.28	>0.001	0.92
Mobility & transport^1^	-0.50	±0.37	-1.35	0.177	0.93
Mobility & transport^2^	-0.22	±0.34	-0.66	0.512	1.06
Resources & consumption^1^	-1.31	±0.40	-3.31	>0.001	1.10
Resources & consumption^2^	0.03	±0.37	0.082	0.934	1.21
Organism^1^	-0.11	± 0.34	0.31	0.754	0.99
Organism^2^	-1.47	± 0.49	-3.03	0.002	0.92
Waste^1^	-1.37	± 0.55	-2.50	0.012	0.81
Waste^2^	-1.49	± 0.39	-3.82	0.001	1.14

eβ = Odds ratio

**Fig 4:**
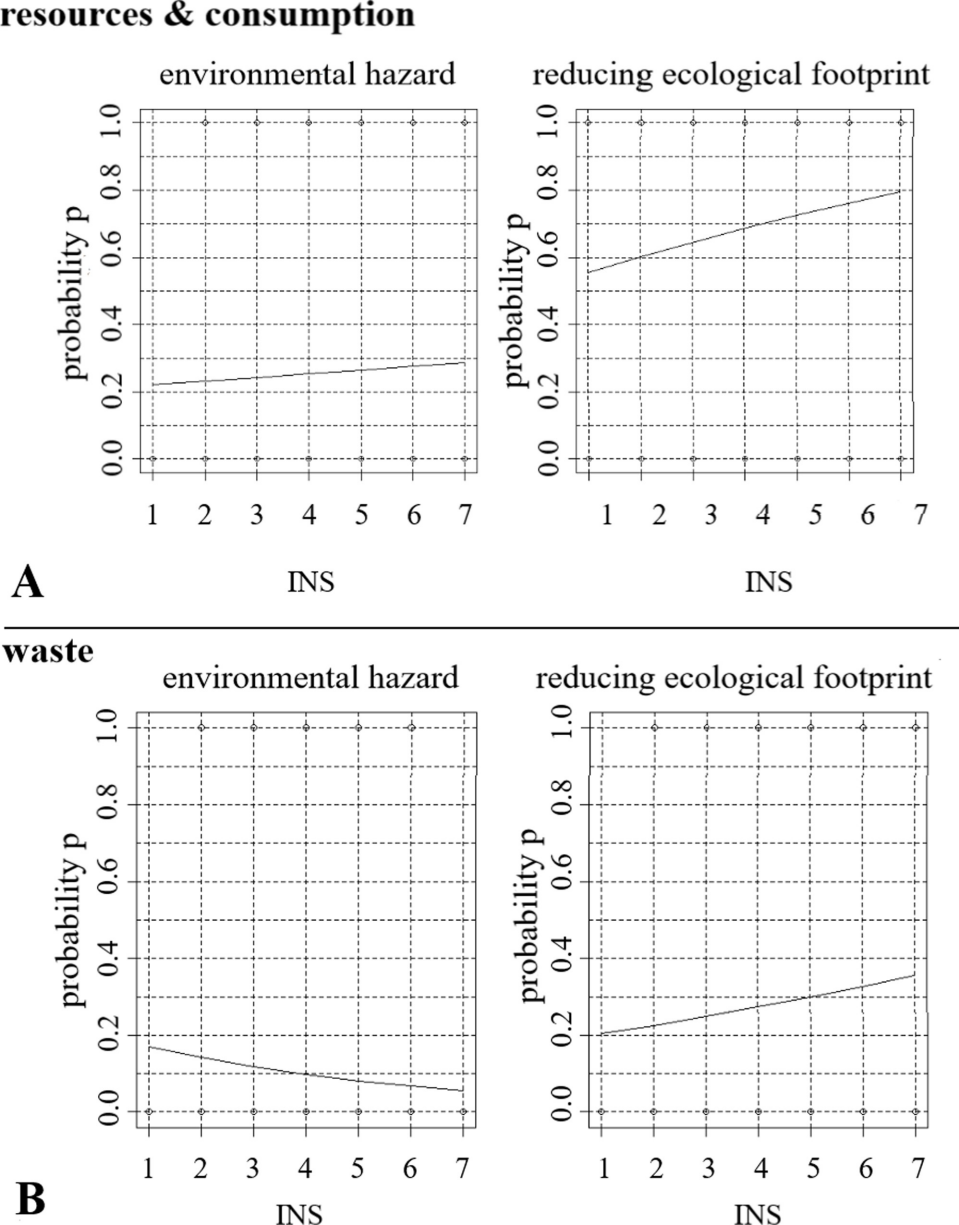
Binary logistic regression analysis, exemplary (A) *resources & consumption* and (B) *waste* perception of the greatest environmental hazard and present ideas to reduce their ecological footprint.

For some categories, the variable INS displayed an approval if a concept was mentioned or not. For the category *resources & consumption*, for example, more concepts were observed if the INS level was higher on both open questions regarding environmental hazard and reducing ecological footprint (Fig 4A). In contrast, for the category of *waste*, less approval follows a higher connectedness to nature level by the question of environmental hazard, whereas a more approval follows a higher connectedness to nature level by the question of reducing ecological footprint (Fig 4B).

## Discussion

Environmental ethics aims at how humans think about their interaction with nature. It links theory (*e*.*g*. knowledge) and practice (*e*.*g*. experience), which form life-long conceptions [17]. According to the literature, two antagonistic preferences prevail either to protect or to utilise the environment [36,52,53]. In essence, two psychometric measurements were well established in the 1990s to measure both. Whereas the *New Environmental Paradigm* (NEP) was developed as a one-dimension scale for adults [54], the, *Two Major Environmental Value Model* (2-MEV) assesses two higher-order factors (preservation, utilization and appreciation of nature) to identify both values for adults and adolescents [52,55]. We decided to integrate the measuring instrument of the *Inclusion of Nature in Self* (INS) scale [56] as a reference-value for connectedness to nature. Concerning the freshmen’s perceptions of our previous study, which was based on closed questions present a clear result: The connectedness to nature level [48] to out a tendency towards an anthropocentric self-perception for all participants (*M* = 3.954, *SD* ± 1.15) in comparison to a human-perception of an ecocentric worldview as ideal (*M* = 5.024, *SD* ± 1.17) [47]. We used this variable as a independent variable for binary logistic regression analysis. Differences of observation were found between some categories of the greatest environmental hazards and concept ideas of reducing the freshmen’s ecological footprint categories.

### Environment and nature

For the environment, the overall conceptions display a range of scientific concepts including human perception. This is not in line with a study of adolescents (13–14 years old), where no human dominance was observed [57]. Half of them followed the idea of nature, a finding that other studies with adults confirmed [58,59]. According to literature, the *environment* was associated in the 1990s with degradation [60] whereas in our study only a few conceptions concerning anthropocentric influences (*e*.*g*. town) were observed. In contrast, *nature* is perceived as almost entirely positive in itself, in the present and the past. Regarding the most observed main categories concerning *emotion/feelings* toward nature, we identified three sub-categories: calmness (*N* = 277), joy (*N* = 149) and aesthetic appreciation (*N* = 129). This stands for a good, self-determined life, a symbol of nature as a good life and a place for relaxation [61]. Most observations referred to freedom/free and silence. This is in line with more than thousands of young people, who associated the concept of nature with peace, recreation, forest, beauty, animals and plants [62]. Nature, in general, is often accompanied by beautiful childhood memories [63]. How can we protect the environment when some concepts are missing or misunderstood? Our study findings revealed little information about perceptions of animals, plants, organism or humans. No conceptions relating to fungi, microorganisms or bacteria were observed. Fewer findings referred to ecological threats and less interest (adapted from the previous study [47]), detected by the same participants. Furthermore, it omitted that conceptions regarding the term *environment* by a view and self-interest regarding the subjective theory. As already outlined, conceptions are perceived to depend on the topic. Values reflect intrinsic motivation [64] to protect the environment. However, social desirability rises with increasing age, which is confirmed by numerous studies (*e*.*g*. [65–67]). Several authors criticize the shift toward sustainable development. Bonnet, for example, rejected the sustainable concept of a human-related relationship with nature [57]. The Brundtland Report (known as Our Common Future) fosters sustainability first [4], following the Rio Declaration of Agenda 2021 and 2030 [6,68]. An appropriate ecocentric education possibility is necessary because it includes all lifeforms and ecosystems with its intrinsic value [69]. This is significant because human welfare concerning ESD is positioned in the centre [70,71]. We concluded that is not important whether perceptions of the environment are following just one ethical concept. It is more important how many conceptions are available in accordance with a persons' prevalent values-system. Here, we confirm self-interest regarding feelings and emotions. However, the environment was under a wide range of humans as a part of it.

### Environmental hazard versus ecological footprint perceptions

The *Club of Rome* became one of the first publications pointing at the limitations of sustainability [2]. Further documents highlighted the planetary carrying capacity as affected by natural resources (soil, air, water and genetic diversity) [3]. Climate change, microplastic, light pollution, species extinction–the list of problems that are endangering the nature and environment, seems endless. How do people perceive threats to the environment and in what sense are humans willing to interact with nature? Concept ideas concerning the greatest environmental hazards and perceptions to reduce freshmen's’ ecological footprints were not perceived equal. By using the connectedness to nature level as an explanatory variable, some categories showed no effect, but others do. Almost two hundred freshmen perceptions referred to the human as the greatest environmental hazard threat, independent of their declared value of connectedness to nature. One of the greatest threats concerns the sub-category *air and land pollution* (*N* = 112). The most important result regarding the ecological footprint was the mean category *resource and consumption*. Many conceptions were found regarding *saving energy and water* (*N* = 163), resources & *consumption* (*e*.*g*. fair trade, second hand and regional/ seasonal) (*N* = 150) and *food consumption* (*N* = 107). Regional and seasonal components seem to be the first step towards sustainability in the freshmen's' minds if they implement their concept idea in their daily life. According to various studies, humans work hard to change their habits in general [72,73]. The following study about consumer perception, awareness of meat production and consumer willingness of changing behaviour regarding sustainable protein consumption (alternatives, insects) was not received well [74]. Our present study findings pointed to a variety of conceptions regarding the main category *waste* and *mobility & transport*. One relevant sub-category refers to *restrict mobility* (*N* = 110) and *car/aircraft/ship* (*N* = 111). Similarities follow using more *public transport/riding a bicycle/carpool* (*N* = 157), which was not surprising if the university was in the city centre and parking lots possibilities are rare and expensive. Based on the participants declare value of connectedness to nature, the higher the INS was, the higher they scored in each of the three categories, which is *waste*, *mobility & transport* and *resources & consumption*. Intervention in environmental studies have shown that the effect of improving the connectedness to nature level contributes to an environmentally friendly consumption behaviour [75]. A suggestion would be add a reliable psychological measuring instrument [76] to explore patterns in conception and behaviour equally. A considerable proportion of variance is unpredictable (e.g. social desirability, self-interest) [77] that we cannot exclude in our data.

## Conclusion

Sustainable perceptions are present in freshmen minds after having completed primary and secondary school within the past UN decade. As a result, half of all responses expressed ideas of fair trade, second hand and regional/ seasonal products. A similar pattern applies to saving energy and water resources. Alternatives to mobility and transport were often stated though perceptions and conscious implementation of concept ideas still requires disentanglement. General scientific concepts were present for the term environment (e.g. interaction between organism or habitat), which integrated humans as a part of it and as one of the greatest environmental hazards. The freshmen responses predominantly showed a self-perception as being a part of the environment (we live/surround us) against a small group in the centre, (I live/surround me). Many perceptions about the environment refer to nature associated with positive emotions and feelings (e.g. hobby, calmness, relaxation). However, ESD creates conceptions aligned with danger and ideas for less exploitation of natural resources although concerning human prosperity. Green educational initiatives have shown that individual behaviour can be influenced positively in the course outreach modules, which has also been shown at the outreach facility Biosphere 2; there, students not only gained system knowledge based on an informal half-day educational program, the latter also induced changes in motivation or fascination, which affect behaviour accordingly [78]. Furthermore, a classroom project demonstrated that energy consumption can be reduced within a ten-week intervention based on a daily routine to prompt environmentally friendly behaviour [79]. In this case, students who demonstrate lower environmental behaviour scores increase their knowledge (action-related and effectiveness knowledge) to the same level as those with higher scores. Future studies concerning the ESD goals may need to focus on qualitative and quantitative conceptions and improve educational interaction in general.

## Supporting information

S1 Dataset Dataset of environment and nature. (XLSX)

## References

[1] Carson R. Silent Spring. Boston, MA: Houghton Mifflin Co.; 1962.

[2] Meadows DH, Meadows DL, Randers J, Behrens WW. The limits to growth. New York; 1972.

[3] Boylan M. Environmental Ethics Second Edi. Boylan M, editor. Encyclopedia of Biodiversity. Wiley-Blackwell; 2013 10.1016/b0-12-226865-2/00106-1

[4] UNWCED (United Nations World Commission on Environment and Development). Our Common Future (Brundtland Report). Oxford: Oxford University Press, UK; 1987.

[5] UNESCO-IEEP. Environmental Education: Module for Pre-Service Training of Science Teachers and Supervisors for Secondary Schools. Environmental Educational Series. 1985. p. 123.

[6] Nation United. Rio Declaration on Environment and Development—Preamble 3rd ed. In: Wheeler SM, Beatley T, editors. The Sustainable Urban Development Reader. 3rd ed. Taylor & Francis; 1992 pp. 79–86.

[7] UNESCO-UNEP. Environmental Literacy. Connect. 1989: 1–2. Available: http://unesdoc.unesco.org/images/0015/001535/153577eo.pdf

[8] UNESCO. The Stockholm Declaration. Stockholm, Sweden; 1972.

[9] UNESCO UNEP. Recommendations of the Intergovernmental Conference on Environmental Education Tbilisi. USSR. France: UNESCO; 1978. pp. 1–96.

[10] UN (United Nation). The Sustainable Development Goals Report 2017. New York; 2017.

[11] Rieckmann M. Education for Sustainable Development Goals: Learning Objectives. Paris, France: UNESCO; 2017.

[12] Bogner FX, Wiseman M. Outdoor ecology education and pupils’ environmental perception in Preservation and Utilisation. Sci Educ Int. 2004;15: 27–48.

[13] Kollmuss A, Agyeman J. Mind the Gap: Why do people act environmentally and what are the barriers to pro-environmental behaviour? Environ Educ Res. 2002;8: 239–260. 10.1080/13504620220145401

[14] Andresen L, Boud D, Cohen R. Experience-based learning Foley G (Ed), Understandisng adult education and training (2nd ed, pp 225–239). Sydney: Allen & Unwin.; 1999 10.1016/j.nepr.2008.05.002

[15] Piaget J. Cognitive development in children: Piaget development and learning. J re. 1964;2: 176–186.

[16] Kaiser FG, Hartig T, Brügger A, Duvier C. Environmental Protection and Nature as Distinct Attitudinal Objects: An Application of the Campbell Paradigm. Environ Behav. 2011;45: 369–398. 10.1177/0013916511422444

[17] DeKock A, Sleegers P, Voeten MJM. New Learning and the Classification of Learning. Rev Educ Res. 2004;74: 141–170. 10.3102/00346543074002141

[18] Buell L. The future of environmental criticism: Environmental crisis and literary imagination. Blackwell Publishing; 2009.

[19] Manni A, Sporre K, Ottander C. Mapping What Young Students Understand and Value Regarding Sustainable development. Int Electron J Environ Educ. 2013;3: 17–35.

[20] Gitay H, Suárez A, Watson R. Climate change and biodiversity. IPCC Rep. 2002; 77 10.2307/1551672

[21] Thorn CJ, Bissinger K, Thorn S, Bogner FX. ‘Trees live on soil and sunshine!’—Coexistence of scientific and alternative conception of tree assimilation. PLoS One. 2016;11 10.1371/journal.pone.0147802 26807974 PMC4725716

[22] Gungordu N, Yalcin-Celik A, Kilic Z. Students’ Misconceptions about the Ozone Layer and the Effect of Internet-Based Media on It. Int Electron J Environ Educ. 2017;7: 1–16. Available: http://ezproxy.lib.uconn.edu/login?url=https://search.ebscohost.com/login.aspx?direct=true&db=eric&AN=EJ1130607&site=ehost-live

[23] Çimer OS, Çimer A, Ursavas N. Student teachers’ conceptions about global warming and changes in their conceptions during pre-service education: a cross sectional study. Educ Res Rev. 2011;6: 592–597.

[24] Bonnett M, Elliot J. Editorial. Cambridge J Educ. 1999;29: 309–311.

[25] Pelletier LG, Tuson KM, Green-Demers I, Noels K, Beaton AM. Why Are You Doing Things for the Environment? The Motivation Toward the Environment Scale (MTES). J Appl Soc Psychol. 1998;28: 437–468. 10.1111/j.1559-1816.1998.tb01714.x

[26] Quinn F, Castéra J, Clément P. Teachers’ conceptions of the environment: anthropocentrism, non-anthropocentrism, anthropomorphism and the place of nature. Environ Educ Res. 2016;22: 893–917. 10.1080/13504622.2015.1076767

[27] Payne P. Children’s Conceptions of Nature. Aust J Environ Educ. 1998; 19–26.

[28] Castéra J, Clément P, Munoz F, Bogner FX. How teachers’ attitudes on GMO relate to their environmental values. J Environ Psychol. 2018;57: 1–9.

[29] Taylor PW. Respect for nature: A theory of environmental ethics. Environmental Ethics. 2013 pp. 152–162.

[30] Ingold T. The Perception of the Environment: Essays on Livelihood, Dwelling and Skill. 1std Editi. Routledge; 2002.

[31] VonUexkúll JJ. The theory of meaning. Simiotica. 1982;42: 25–82.

[32] Lévêque C. Ecology From Ecosystem to Biosphere. CRC Press; 2003.

[33] Ingold T. Culture and the perception of the environment In: Croll E, Parkin D, editors. Bush Base, Forest Farm. 2014 10.4324/9780203036129

[34] Attfield R. Environmental ethics: An overview for the twenty-first century. second edi. John Wiley & Sons; 2014.

[35] Bogner FX, Brengelmann JC, Wiseman M. Risk-taking and environmental perception. Environmentalist. 2000;20: 49–62.

[36] Cedillo CV. On Empathy, Anthropocentrism, and Rhetorical Tropes: An Analysis of Online “Save the Bees!” Campaign Images. Screening the Nonhuman: Representations of Animal Others in the Media. 2016. p. 185.

[37] Botar O. Defining Biocentrism. Biocentrism Mod. 2017; 15–46.

[38] Thompson SCG, Barton MA. Ecocentric and Anthropocentric Attitudes Toward the Environment. J Environ Psychol. 1994;14: 149–157.

[39] O’neill J, Holland A, Light A. Environmental values. Abingdon, Routledge; 2008.

[40] Bogner FX, Wiseman M. Toward Measuring Adolescent Environmental Perception. Eur Psychol. 1999;4: 139–151. 10.1027//1016-9040.4.3.139

[41] Schneiderhan-Opel J, Bogner FX. Between environmental utilization and protection: Adolescent conceptions of biodiversity. Sustainability. 2019;11 10.3390/su11174517

[42] Schneiderhan-Opel J, Bogner FX. The relation between knowledge acquisition and environmental values within the scope of a biodiversity educational module. Sustainability. 2020;12 10.3390/su12062323

[43] Frantz CMP, Mayer FS. The importance of connection to nature in assessing environmental education programs. Stud Educ Eval. 2014;41: 85–89. 10.1016/j.stueduc.2013.10.001

[44] Otto S, Pensini P. Nature-based environmental education of children: Environmental knowledge and connectedness to nature, together, are related to ecological behaviour. Glob Environ Chang. 2017;47: 88–94. 10.1016/j.gloenvcha.2017.09.009

[45] Blumstein DT, Saylan C. The failure of environmental education (and how we can fix it). PLOSBiology. 2007;5 10.1371/journal.pbio.0050120 17439304 PMC1847843

[46] Worldbank. 2020 [cited 26 Feb 2020]. Available: https://data.worldbank.org/indicator/EN.POP.DNST?end=2018&locations=CH&start=1997&year_high_desc=false

[47] Maurer M, Bogner FX. How freshmen perceive environmental education (EE) and education for sustainable development (ESD). PLoS One. 2019;14: 1–16. 10.1371/journal.pone.0208910 30640908 PMC6331178

[48] Liefländer AK, Fröhlich G, Bogner FX, Schultz PW. Promoting connectedness with nature through environmental education. J Environ Educ. 2013;19: 370–384. 10.1080/13504622.2012.697545

[49] Mayring P. Qualitative Content Analysis. Qual Soc Res. 2000;1.

[50] Cohen J. A coefficient for agreement for nominal scales. Educ Psychol Meas. 1960;20: 37–46.

[51] Suzuki R, Shimodaira H. Pvclust: an R package for assessing the uncertainty in hierarchical clustering. Bioinformatics. 2006;22: 1540–1542. 10.1093/bioinformatics/btl117 16595560

[52] Wiseman M, Bogner FX. A higher-order model of ecological values and its relationship to personality. Pers Individ Dif. 2003;34: 783–794.

[53] Bogner FX, Wiseman M. Adolescents’ attitudes towards nature and environment: Quantifying the 2-MEV model. Environmentalist. 2006;26: 247–254. 10.1007/s10669-006-8660-9

[54] Dunlap RE, Van Liere KD. The “new environmental paradigm”. J Environ Educ. 1978;9: 10–19.

[55] Bogner FX. Environmental values (2-MEV) and appreciation of nature. Sustainability. 2018;10 10.3390/su10020350

[56] Schultz. Inclusion with nature: Understanding the psychology of human–nature interactions. In: Schultz. In P. Schmuck & P. W., (Eds.), Development T psychology of sustainable, New (pp. 61–78)., Kluwer. Y, editors. Psychology of sustainable development. Boston; 2002. pp. 61–78.

[57] Pointon P. ‘The city snuffs out nature’: young people’s conceptions of and relationship with nature. Environ Educ Res. 2014;20: 776–794. 10.1080/13504622.2013.833595

[58] Munoz F, Bogner F, Clement P, Carvalho GS. Teachers’ conceptions of nature and environment in 16 countries. J Environ Psychol. 2009;29: 407–413. 10.1016/j.jenvp.2009.05.007

[59] Flogaitis E, Agelidou E. Kindergarten teachers’ conceptions about nature and the environment. Environ Educ Res. 2003;9: 461–478.

[60] Gerhard T. Die Geschichte ökologisch bedeutsamer Naturvorstellungen in deutschen Bildungskonzepten. Weinheim; 1990.

[61] Kellert SR. The biological basis for human values of nature In: Washington D., editor. The biophilia hypothesis. Island Press; 1993 pp. 42–69.

[62] Schuster K, Hartkemeyer T, Krömker D. Naturschutzorientierte Lebensstilorientierungen bei Jugendlichen [Nature conservation-oriented lifestyle orientations among students]. In: Schuster K., Hartkemeyer T., Krömker D, editor. Gesellschaft und Naturschutz. Bonn, Bad Godesberg, Germany: Bad Godesberg; 2008 pp. 89–92.

[63] Strife S, Downey L. Childhood development and access to nature: A new direction for environmental inequality research. Organ Environ. 2009;22: 99–122. 10.1177/1086026609333340 21874103 PMC3162362

[64] Steg L, De Groot JI. Environmental values. The Oxford handbook of environmental and conservation psychology 2012.

[65] Kaiser FG. A general measure of ecological behavior. J Appl Soc Psychol. 1998;28: 395–422. 10.1111/j.1559-1816.1998.tb01712.x

[66] Boeve-de Pauw J, Van Petegem P. Because my friends insist or because it makes sense? Motivation towards the Environment. Sustainability. 2017;9 10.3390/su9050750

[67] Oerke B, Bogner FX. Gender, age and subject matter: impact on teachers’ ecological values. Environmentalist. 2010;30: 111–122.

[68] UNGA. Transforming our world: The 2030 agenda for sustainable development A New Era Glob Heal. New York, NY: UN General Assembly; 2015.

[69] Washington H, Taylor B, Kopnina H, Cryer P, Piccolo JJ. Why ecocentrism is the key pathway to sustainability Environmental education (EE) View project. 2017;1: 35–41.

[70] Kopnina H. Education for the future? Critical evaluation of education for sustainable development goals. J Environ Educ. 2020; 1–12. 10.1080/00958964.2019.1710444

[71] Kopnina H. Education for sustainable development (ESD): The turn away from ‘environment’ in environmental education? Environ Educ Res. 2012;18: 699–717. 10.1080/13504622.2012.658028

[72] Henn L, Taube O, Kaiser FG. The role of environmental attitude in the efficacy of smart-meter-based feedback interventions. J Environ Psychol. 2019;63: 74–81. 10.1016/j.jenvp.2019.04.007

[73] Rieger A, Thummert R, Fridgen G, Kahlen M, Ketter W. Estimating the benefits of cooperation in a residential microgrid: A data-driven approach. Appl Energy. 2016;180: 130–141. 10.1016/j.apenergy.2016.07.105

[74] Hartmann C, Siegrist M. Consumer perception and behaviour regarding sustainable protein consumption: A systematic review. rends Food Sci Technol. 2017;61: 11–25.

[75] Fröhlich G, Sellmann D, Bogner FX. The influence of situational emotions on the intention for sustainable consumer behaviour in a student-centred intervention. Environ Educ Res. 2013;19: 747–764. 10.1080/13504622.2012.749977

[76] Kaiser FG, Oerke B, Bogner FX. Behavior-based environmental attitude: Development of an instrument for adolescents. J Environ Psychol. 2007;27: 242–251. 10.1016/j.jenvp.2007.06.004

[77] Kaiser FG, Hübner G, Bogner FX. Contrasting the Theory of Planned Behavior With the Value-Belief-Norm Model in Explaining Conservation Behavior. J Appl Soc Psychol. 2005;35: 2150–2170.

[78] Baierl T, Bonine K, Johnson B, Bogner FX. Effects of informal learning on science motivation, fascination, and system knowledge at Biosphere 2.

[79] Maurer M, Koulouris P, Bogner FX. Green Awareness in Action—How Energy Conservation Action Forces on Environmental Knowledge, Values and Behaviour in Adolescents’ School Life. Sustainability. 2020;12: 955 10.3390/su12030955

